# Supplementary material for: Brainstem neurons coordinate the bladder and urethral sphincter for urination
Source: eLife. 2026 Aug 6;13:RP103224. doi: 10.7554/eLife.103224 (PMC13446904; doi:10.7554/eLife.103224)
Supplement: Peer review history

# Editor report: eLife Assessment

**DOI:** 10.7554/eLife.103224.3.sa0

**Authors:** Timothy E Behrens

**Affiliations:** University of Oxford United Kingdom

**Keywords:** Convincing, Important

In this **important** study, Li et al. identify estrogen receptor 1-expressing neurons (ESR1+) in Barrington's nucleus as key regulators coordinating both bladder contraction and the relaxation of the external urethral sphincter. Using appropriate and validated methodologies aligned with the current state of the art, the data are **convincing** and of generally high quality.

# Referee report: Reviewer #1 (Public review)

**DOI:** 10.7554/eLife.103224.3.sa1

[Editor's note: this version has been assessed by the Reviewing Editor with further input from the original reviewers. The authors have addressed the comments raised in the previous round of review.]

Summary:

Urination requires precise coordination between the bladder and external urethral sphincter (EUS), while the neural substrates controlling this coordination remain poorly understood. In this study, Li et al. identify estrogen receptor 1-expressing neurons (ESR1+) in Barrington's nucleus as key regulators that faithfully initiate or suspend urination. Results from peripheral nerve lesions suggest that BarEsr1 neurons play independent roles in controlling bladder contraction and relaxation of the EUS. Finally, the authors performed region-specific retrograde tracing, claiming that distinct populations of BarEsr1 neurons target specific spinal nuclei involved in regulating the bladder and EUS, respectively.

Strength:

Overall, the work is done with high quality. The authors integrate several cutting-edge technologies and sophisticated, thorough analyses, including opto-tagged single unit recordings, combined optogenetics and urodynamics, particularly those following distinct peripheral nerve lesions.

Comments on revised version:

During the revision, the authors have adequately addressed my concerns and made the suggested changes accordingly. I have no additional comments.

# Referee report: Reviewer #2 (Public review)

**DOI:** 10.7554/eLife.103224.3.sa2

Summary:

The authors have performed a rigorous study to assess the role of ESR1+ neurons in the PMC to control coordination of bladder and sphincter muscles during urination. This is an extension of previous work defining the role of these brainstem neurons, and convincingly adds to the understanding of their role as master regulators of urination. This is a thorough, well-done study that clarifies how the Pontine micturition center coordinates different muscle groups for efficient urination, but there are some questions and considerations that remain.

Strengths:

These data are thorough and convincing in showing that ESR1+ PMC neurons exert coordinated control over both the bladder and sphincter activity, which is essential for efficient urination. The anatomical distinctions in pelvic versus pudendal control is clear, and it's an advance to understand how this coordination occurs. This work offers a clearer picture of how micturition is driven.

Weaknesses:

The dynamics of how this population of ESR1+ neurons is engaged in natural urination events remains unclear. Not all ESR1+neurons are always engaged, and it is not measured whether this is simply variation in population activity, or if more neurons are engaged during more intense starting bladder pressures, for instance. In particular, the response dynamics of single and doubly-projecting neurons are not defined. Additionally, the model for how these neurons coordinate with CRH+ neuron activity in the PMC is not addressed, although these cell types seem to be engaged at the same time. Lastly, it would be interesting to know how sensory input can likely modulate the activity of these neurons, but this is perhaps a future direction.

# Referee report: Reviewer #3 (Public review)

**DOI:** 10.7554/eLife.103224.3.sa3

Summary:

The paper by Li et al explored the role of Estrogen receptor 1 (Esr1) expressing neurons in the pontine micturition center (PMC), a brainstem region also known as Barrington's nucleus (Hou wt al 2016, Keller et al 2018). First the author conducted bulk Ca2+ imaging/unit recording from PMCESR1 to investigate the correlations of PMCESR1 neural activity to voiding behavior in conscious mice and bladder pressure/external urethral muscle activity in urethane anesthetized mice. Next the authors conducted optogenetics inactivation/activation of PMCESR1 to confirm the contribution to the voiding behavior also conducted peripheral nerve transection together with optogenetics activation to confirm the independent control of bladder pressure and urethral sphincter muscle.

Comments on revised version:

No concerns. All my major questions were addressed.

# Author comment: Author response

**DOI:** 10.7554/eLife.103224.3.sa4

**Authors:** Xing Li, Xianping Li, Jun Li, Han Qin, Shanshan Liang, Jun Li, Tingliang Jian, Xia Wang, Lingxuan Yin, Chunhui Yuan, Xiang Liao, Hongbo Jia, Xiaowei Chen, Jiwei Yao

**Affiliations:** Guangxi University Nanning China; Second Military Medical University Shanghai China; Guangxi University Guangxi China; Chongqing Institute for Brain and Intelligence Chongqing China; Army Medical University Chongqing China; Army Medical University Chongqing China; Army Medical University Chongqing China; Chongqing University Chongqing China; Guangxi University Guangxi China; Second Military Medical University Shanghai China; Chongqing University chongqing China; Suzhou Institute of Biomedical Engineering and Technology, Chinese Academy of Sciences Suzhou China; Army Medical University Chongqing China; Second Military Medical University Shanghai China

The following is the authors’ response to the original reviews.

We would like to express our deep appreciation to the editor and reviewers for their constructive comments and suggestions, which have significantly improved the quality of our manuscript. In response, we have carefully revised the manuscript, addressed all comments, and performed additional experiments and analyses to strengthen our findings.

(1) We repeated retrograde tracing using CTB-647 to verify precise targeting of SPN and DGC neurons, as shown in the new Figure 7.

(2) We performed dual retrograde tracing combined with fiber photometry or optogenetic activation to investigate the role of PMC dual-projecting neurons in the control of urination, as shown in Figure supplements 11 and 12.

(3) We conducted new experiments activating PMC^ESR1+^ neurons after PDNx to assess their role in urination, as shown in new Figure 6.

(4) We added a more detailed analysis of the dynamics of neural responses in PMC^ESR1+^ neurons in Figure supplements 3F-3G.

(5) We analyzed peak Ca^2+^ signals in the PMC during and after the onset of EMG bursting, as shown in Figure supplement 4F.

(6) We added a comparison of spontaneous and light-induced spikes in PMC^ESR1+^ neurons, as shown in Figure supplements 3B–3C.

(7) We expanded the Discussion to address how PMC^ESR1+^ neurons coordinate bladder contraction and sphincter relaxation to control both the initiation and suspension of urination.

We hope these revisions meet the reviewers' expectations and contribute to the improvement of our manuscript.

**Reviewer #1 (Public review):**

Summary:

Urination requires precise coordination between the bladder and external urethral sphincter (EUS), while the neural substrates controlling this coordination remain poorly understood. In this study, Li et al. identify estrogen receptor 1-expressing neurons (ESR1+) in Barrington's nucleus as key regulators that faithfully initiate or suspend urination. Results from peripheral nerve lesions suggest that BarEsr1 neurons play independent roles in controlling bladder contraction and relaxation of the EUS. Finally, the authors performed region-specific retrograde tracing, claiming that distinct populations of BarEsr1 neurons target specific spinal nuclei involved in regulating the bladder and EUS, respectively.

Strengths:

Overall, the work is of high quality. The authors integrate several cutting-edge technologies and sophisticated, thorough analyses, including opto-tagged single unit recordings, combined optogenetics, and urodynamics, particularly those following distinct peripheral nerve lesions.

We are grateful for your insightful and constructive comments, which affirmed the importance and technical depth of our work. Thank you for dedicating your expertise and time to reviewing our manuscript. Guided by your suggestions, we have revised the paper as detailed below.

Weaknesses:

(1) My major concern is the novelty of this study. Keller et al. 2018 have shown that BarEsr1 neurons are active during urination and play an essential role in relaxing the external urethral sphincter (EUS). Minimally, substantial content that merely confirms previous findings (e.g. Figures 1A-E; Figures 3A-E) should be move to the supplementary datasets.

Thank you for this valuable and constructive comment. We fully agree that the novelty of our study relative to Keller et al., 2018 must be made explicit. Keller et al. established that PMC^ESR1+^ neurons are active during socially evoked urine-marking behavior (voluntary urination) and demonstrated their essential role in relaxing the EUS. Their study mainly focused on behavioral context and EUS relaxation. In contrast, our work addresses a distinct, mechanistic question: how these same neurons participate in reflexive, physiological urination and coordinate both bladder detrusor contraction and EUS relaxation.

Novel aspects of the present study:

(1) Temporal dynamics of PMC^ESR1+^ neurons during reflexive micturition.

Using opto-tagging and single-unit recordings, we reveal the precise firing pattern of PMC^ESR1+^ neurons during reflexive voiding. Simultaneous fiber photometry, cystometry, and EUS-EMG recordings demonstrate that population-level activity of PMC^ESR1+^ neurons precedes and tightly correlates with both bladder contraction and EUS relaxation a coordination not previously demonstrated.

(2) Causal role in reflexive urination.

Manual closed-loop optogenetic inhibition at the onset of reflexive voiding acutely terminates EUS bursting and bladder contraction, immediately halting urine release.

(3) Dual control of bladder and EUS.

Optogenetic activation combined with selective pelvic or pudendal nerve transection shows that PMC^ESR1+^ neurons drive both bladder contraction and EUS relaxation, revealing a coordinating role beyond EUS relaxation alone.

(4) Anatomical substrate for coordinated control of bladder contraction and EUS relaxation in reflexive urination.

Retrograde tracing identifies three spinal-projecting sub-populations: SPN-only, DGC-only, and dual-targeting neurons, providing a circuit-level explanation for the simultaneous control of bladder and EUS.

Following your suggestion, panels that merely replicate Keller et al. (former Figures 1A–1E and Figures 3A–3E) have been moved to new Figure Supplements 1 and 7, respectively, so that the main figures now emphasize the new mechanistic findings.

(2) I also have concerns regarding the results showing that the inactivation of BarEsr1 neurons led to the cessation of EUS muscle firing (Figures 2G and S5C). As shown in the cartoon illustration of Figure 8, spinal projections of BarEsr1 neurons contact interneurons (presumably inhibitory) that innervate motor neurons, which in turn excite the EUS. I would therefore expect that the inactivation of BarEsr1 should shift the EUS firing pattern from phasic (as relaxation) to tonic (removal of relaxation), rather than stopping their firing entirely. Could the authors comment on this and provide potential reasons or mechanisms for this finding?

Thank you for this crucial comment. We apologize that the representative EUS-EMG traces in Figures 2G and S5C were too small to be clearly seen and that the corresponding results description was not sufficiently accurate. We have now replaced these EMG traces with enlarged versions (revised Figures 2G and S5C) and revised the corresponding Results section (lines 184, 197, 340-341). Based on the enlarged traces, we found that acute photoinhibition of PMC^ESR1+^ neurons at the onset of phasic EUS-EMG bursting shifted the EUS firing pattern from large-amplitude phasic bursts to low-amplitude tonic firing. This suggests that ongoing activity of PMC^ESR1+^ neurons is required to maintain phasic EUS bursting. A similar shift from phasic to tonic EUS-EMG activity during optogenetic silencing of PMC^ESR1+^ neurons was reported by Keller et al., 2018 (Figure supplement 8C), confirming the reproducibility of the phenotype. We propose that the potential mechanism of this low-amplitude tonic activity may be mediated in part by a spinal reflex pathway (the guarding reflex) for preventing urination, whereby the loss of PMC^ESR1+^ neurons-mediated supraspinal facilitation reduces inhibition of spinal interneurons, leading to enhanced baseline excitability of EUS motor neurons in response to bladder afferent input during bladder distension (William C. de Groat et al., Comprehensive Physiology. 2015, PMID: 25589273).

(3) Current evidence is insufficient to support the claim that the majority of BarEsr1 neurons innervate the SPN but not DGC. The current spinal images are uninformative, as the fluorescence reflects the distribution of Esr1- or Crh-expressing neurons in the spinal cord, along with descending BarEsr1 or BarCrh axons. Given the close anatomical proximity of these two nuclei, a more thorough histological analysis is required to demonstrate that the spinal injections were accurately confined to either the SPN or the DGC.

Thank you for raising this important concern. To rigorously verify that our spinal injections were confined to either the SPN or the DGC, we performed new retrograde-tracing experiments in ESR1-Cre and CRH-Cre mice. We injected a mixture of AAV-Retro-DIO-mCherry or AAV-Retro-DIO-EGFP with the retrograde tracer CTB-647 specifically into the SPN or DGC (Methods, lines 465-466). Only animals in which CTB-647 fluorescence was strictly limited to the target nucleus, without detectable spread to the adjacent region, were included in the analysis (new Figures 7A and 7E). These results confirm our original observation that PMC^ESR1+^ neurons comprise three distinct spinal-projection subpopulations: one (19.0%) targeting the SPN, one (52.2%) innervating the DGC, and a third (28.8%) projecting to both regions (Results, lines 304–306; new Figures 7F–7H). In addition, the majority of PMC^CRH+^ neurons project to the SPN but not the DGC (new Figures 7B–7D; Results, lines 297–301). We have assembled new Figure 7 using the newly acquired spinal images and the validated data.

**Reviewer #1 (Recommendations for the authors):**

From the abstract: "Anatomically, PMCESR1+ cells possess two subpopulations projecting to either the pelvic or pudendal nerve". I don't think these neurons directly project to either nerve.

Thank you for this precise comment. We apologize for incorrectly stating that PMC^ESR1+^ cells project directly to the pelvic or pudendal nerves. In the revised Abstract (lines 32–36) we have rephrased the sentence to clarify the actual anatomy: “Anatomically, PMC^ESR1+^ neurons consist of three distinct spinal-projection-based subpopulations: one targeting the sacral parasympathetic nucleus (SPN), one innervating the dorsal gray commissure (DGC), and a third that projects to both regions, thereby enforcing the coordination of bladder contraction and sphincter relaxation in a rigid temporal sequence.”. We trust this revision now accurately reflects the anatomical findings.

**Reviewer #2 (Public review):**

Summary:

The authors have performed a rigorous study to assess the role of ESR1+ neurons in the PMC to control the coordination of bladder and sphincter muscles during urination. This is an important extension of previous work defining the role of these brainstem neurons, and convincingly adds to the understanding of their role as master regulators of urination. This is a thorough, well-done study that clarifies how the Pontine micturition center coordinates different muscle groups for efficient urination, but there are some questions and considerations that remain.

Strengths:

These data are thorough and convincing in showing that ESR1+PMC neurons exert coordinated control over both the bladder and sphincter activity, which is essential for efficient urination. The anatomical distinctions in pelvic versus pudendal control are clear, and it's an advance to understand how this coordination occurs. This work offers a clearer picture of how micturition is driven.

We sincerely thank you for highlighting the rigor of our study and for recognizing the advance in understanding how PMC^ESR1+^ neurons exert coordinated, anatomically segregated control over bladder and sphincter. We also appreciate the constructive suggestions that helped us further improve clarity, which we address point-by-point below.

Weaknesses:

The dynamics of how this population of ESR1+ neurons is engaged in natural urination events remains unclear. Not all ESR1+ neurons are always engaged, and it is not measured whether this is simply variation in population activity, or if more neurons are engaged during more intense starting bladder pressures, for instance. In particular, the response dynamics of single and doubly-projecting neurons are not defined. Additionally, the model for how these neurons coordinate with CRH+ neuron activity in the PMC is not addressed, although these cell types seem to be engaged at the same time. Lastly, it would be interesting to know how sensory input can likely modulate the activity of these neurons, but this is perhaps a future direction.

Thank you for this insightful comment. First, we agree that not all ESR1+ neurons are consistently engaged during urination (Figure 1B). Because bladder pressure was not measured during the opto-tagging experiments, we cannot determine whether this reflects trial-to-trial variability in population activity or pressure-dependent recruitment of additional neurons. We speculate that stronger starting bladder pressures may recruit a larger subset of ESR1+ neurons, analogous to graded, pressure-dependent recruitment observed in peripheral sensory neurons (Bruns et al., J Neural Eng. 2011, PMID: 21878706; Marshall et al., Nature. 2020, PMID: 33057202).

Second, using fiber photometry recording and optogenetic activation, we examined the dynamics of dual-projecting neurons in the PMC that were retrogradely labeled from the SPN and DGC. Their activity correlated with bladder contraction and sphincter relaxation, and optogenetic activation sequentially induced these events to trigger urination (see Recommendation #8). Although retrograde labeling captured only a subset of dual-projecting neurons, the results indicate that they coordinate bladder and sphincter activity.

Third, previous studies suggest that PMC^CRH+^ cells are associated with bladder contraction and likely serve as an integration center for context-dependent micturition behavior (Hou et al., Cell. 2016, PMID: 27662084; Ito et al., Elife. 2020, PMID: 32347794). We therefore propose that PMC^CRH+^ cells establish the baseline conditions and contextual readiness for voiding, whereas PMC^ESR1+^ cells act as the executive command to reliably initiate and execute the event.

Finally, we agree that sensory inputs likely modulate PMC^ESR1+^ neuron activity. Although this falls beyond the scope of the present study, it represents an important avenue for future investigation.

**Reviewer #2 (Recommendations for the authors):**

(1) In the introduction, the authors write that Keller 2018 only showed this ESR1 population to induce EUS relaxation, but those results also do show bladder contraction with photostimulation of this population. While the authors' work extends this finding in important ways, this should be acknowledged (line 60).

Thank you for this important correction. We have now revised the Introduction to explicitly acknowledge that stimulation of neurons expressing estrogen receptor 1 (ESR1) in the PMC (PMC^ESR1+^) contributes to sphincter relaxation and increased bladder pressure (Introduction, lines 60-62), as originally reported by Keller et al., 2018.

(2) I think a more detailed analysis of the dynamics of neural responses in the PMC ESR1 neurons would be valuable. For example: are the same cells always engaged before micturition, or do different populations activate on different trials? Can the authors comment on the half of the opto-tagged ESR1 population that is not firing during urination? Do they ever fire? A cell-by-cell analysis of which neurons are engaged over multiple trials would be very valuable to understand the dynamics of population activity. Figure 1H shows cumulative sessions, but what do single sessions look like?

Thank you for these valuable comments. In response, we have performed refined single-trial analyses of neuronal activity, as detailed in the point-by-point replies below.

For example: are the same cells always engaged before micturition, or do different populations activate on different trials?

Among 11 PMC^ESR1+^ units that showed urination-related excitation, 8 units exhibited a consistent firing increase in every voiding trial, whereas the remaining 3 increased their discharge in >78 % of trials (Figure 1B; new Figure supplement 3F). Thus, the same PMC^ESR1+^ cells are recruited repeatedly, rather than distinct populations being activated on different trials. We have added this clarification to Results (lines 106–108).

Can the authors comment on the half of the opto-tagged ESR1 population that is not firing during urination? Do they ever fire? A cell-by-cell analysis of which neurons are engaged over multiple trials would be very valuable to understand the dynamics of population activity.

Approximately half of the opto-tagged PMC^ESR1+^ cells showed no increase in firing rate during urination, yet exhibited spontaneous spikes at other times (new Figure supplement 3G), confirming their electrical competence. Because the PMC also participates in defecation, uterine activity, and other pelvic functions (Rouzade-Dominguez et al., Eur J Neurosci. 2003, PMID: 14686905; Schellino et al., Frontiers in Neuroanatomy. 2020, PMID: 33013330; Quaghebeur et al., Auton Neurosci. 2021, PMID: 34391125), these ESR1+ neurons may serve functions other than urination. We have now added this cell-by-cell analysis and discussion to the manuscript (Results, lines 108-112).

Figure 1 H shows cumulative sessions, but what do single sessions look like?

As shown in new Figure supplements 3F–3G, single-session raster plots reveal that PMC^ESR1+^ neurons display consistent firing patterns across individual trials. Neurons whose firing rate increased during urination did so in most trials (Figure supplement 3F), whereas neurons unrelated to voiding remained silent or showed no discernible rate change during voiding across trials (Figure supplement 3G). These single-session observations are consistent with the cumulative population analysis shown in Figure 1H (new Figure 1B).

(3) Supplemental Figure 4: It seems clear from this figure that NVCs are only occurring when the sphincter fails to engage. Can the authors quantify how often this is the case?

Thank you for this important point. We have now quantified the occurrence of non-voiding contractions (NVCs) across all 229 bladder contraction events from 3 mice shown in Supplemental Figure 4. NVCs were observed exclusively when the external urethral sphincter failed to relax, accounting for 62/229 events (27.1 %), whereas coordinated voiding contractions (VCs) occurred in the remaining 167 events (72.9 %). These new data are presented in Figure supplement 4C.

(4) Continuing from the above point: the authors say that the insufficient top-down drive or strength of activity from PMC ESR1 neurons is why NVCs occur. In looking closely, it also seems there is a small hump and subsequent increase in the calcium signal when the EUS bursting begins (particularly clear in Supplementary Figure 4). Could this instead mean that the bursting/urethral activity itself is feeding back onto the PMC to continue/enhance its activity, and it is instead the lack of sphincter bursting that results in the NVC? Could the authors analyze the signal during and after bursting starts? This model is consistent with one of the classic reflexes defined by Barrington, in which urethral fluid flow/activation enhances bladder contraction. The Figure 4 transection experiments do not fully answer this, as the authors are driving activity in the PMC at this time, but they could test this using PDN transection with fiber photometry recording.

Thank you for this important point. We fully agree that EUS bursting may provide excitatory feedback to the PMC that sustains or even amplifies its activity, and that the absence of such feedback could underlie NVCs. To test this possibility, we re-analyzed the fiber-photometry traces aligned to the onset and offset of each EUS bursting (new Figure supplement 4). A small but consistent hump in the Ca^2+^ signal appeared before bursting onset and the Ca^2+^ signal continued to rise throughout the bursting (Figure supplement 4B, yellow arrow). The amplitude at bursting offset was significantly higher than both the NVC peak and the level recorded at bursting onset. These observations support the interpretation that urethral fluid flow/activation supplies excitatory feedback that reinforces PMC activity and bladder contraction, consistent with Barrington’s classic reflex. We have incorporated these new analyses into the revised manuscript (lines 145–155 and Figure supplement 4F).

We agree that the positive-feedback loop described by Barrington’s classic urethra-to-bladder reflex is an intriguing mechanism. However, the PDN-transection experiment in Figure 4 was designed to determine if bladder contractions triggered by PMC^ESR1+^ cells can proceed in the absence of sphincter bursting, not to evaluate this reflex. Incorporating simultaneous fiber-photometry recording into the PDN-transection experiment would therefore go beyond the scope of the present study. In future work we are keen to combine PDN transection with fiber photometry to further determine whether the urethra-to-bladder reflex contributes to the sustained PMC activity observed in our paradigm.

(5) In Figure 4, is the timing of sphincter engagement different with ChR2 stimulation from what normally occurs? It appears that the bursting happens immediately upon activation whereas bladder contraction is a bit delayed.

Thank you for this important observation. We have carefully re-examined the EMG traces from all animals shown in Figure 4. We confirm that the onset of sphincter bursting activity during ChR2 stimulation is indeed more rapid than during natural reflex voiding; nevertheless, the onset of phasic sphincter bursting during ChR2 stimulation remained delayed relative to the intravesical pressure rise (see Figure 8B).

The immediate sphincter discharge visible in some trials was tonic EUS discharge or rare irregular bursting, not the typical EUS bursting. This tonic pattern corresponds to the spinal guarding reflex that suppresses urine leakage (Fowler et al., Nature Reviews Neuroscience. 2008, PMID: 18490916; Keller et al., Nature Neuroscience. 2018, PMID: 30104734). These segments were identified by their amplitude and spectral content and excluded from burst-onset analysis. Our analysis protocol therefore distinguishes tonic guarding activity from true phasic bursting, ensuring that only the latter was used to determine burst timing.

(6) The explanation on line 299 about how spinal reflexes are impinging on this circuit is confusing. I agree that the bladder contraction stopping later than the EUS signal likely has something to do with spinal reflexes, but it seems this could instead be feedback from the urethral fluid flow, which continues bladder contractions (urethra-destrusor facilitative reflex). Could the authors clarify their thoughts here?

Thank you for highlighting this ambiguity. We agree that the delayed cessation of bladder contraction could equally reflect either (1) the urethra-to-bladder facilitative reflex driven by ongoing urethral fluid flow or (2) spinal reflexes that we described. In the revised manuscript (Results, lines 343–349), we have re-worded the paragraph to make this dual possibility explicit, thereby avoiding an overly strong emphasis on spinal mechanisms alone.

(7) A note on phrasing: the authors frequently say PMCESR1 cells drive sphincter relaxation, but then show an effect on sphincter bursting. Experienced readers might realize that relaxation and bursting are connected, but this might be confusing for readers and should be clarified in the text.

Thank you for highlighting the potential ambiguity. We agree that the sentence “PMC^ESR1^ cells drive sphincter relaxation” can seem paradoxical when our data show increased EUS bursting. In adult mice, the EUS does not remain continuously relaxed during voiding; instead, it generates rhythmic bursting composed of high-frequency spike clusters (active periods) alternating with low tonic activity (silent periods), resulting in rhythmic contractions and relaxations of EUS. This phasic activity acts as a pump that facilitates urine flow through the narrow rodent urethra (Kadekawa et al., Am J Physiol Regul Integr Comp Physiol, 2016, PMID: 26818058). The EUS bursting activity we recorded is consistent with the results reported in previous studies (Keller et al., Nat Neurosci, 2018, PMID:30104734; Ito et al., Elife, 2020, PMID:32347794).

Consequently, when PMC^ESR1^ neurons initiate bursting, they simultaneously generate the relaxation phases that separate the spikes. To make this explicit we have replaced the phrase “PMC^ESR1+^ cells drive sphincter relaxation” with “PMC^ESR1^ neurons trigger EUS bursting, which generates rhythmic sphincter contractions and relaxations.” (Results, page 7, lines 219-221). We have applied similar clarifications throughout the revised manuscript (Results, lines 125-129). We hope this revision eliminates any apparent contradiction.

(8) The question remains as to which neurons (dual projecting, single projecting, or all?) are active in natural urination. This is possible to do through dual injection of retrograde virus in SPN and DGC that could coordinately turn on Gcamp, but this challenging experiment is perhaps beyond the scope of this paper. Even still, the authors could discuss their model for whether the dual- and single-projecting neurons are all engaged at once in a natural urination event. Do the authors have any data that could provide insight as to when these sub-populations are active? Results from the opto-tagging in Figure 1 (and comment #2 about single neuron firing properties) might provide a foundation for hypotheses or insights.

Thank you for this valuable suggestion. We have now performed the experiment you proposed: dual injection of retrograde virus (AAV-Retro-Cre and AAV-Retro-DIO-GCaMP6s) in SPN and DGC were used to selectively label PMC dual-projecting neurons, and a 200-µm optic fiber was implanted above the PMC to record their Ca^2+^ dynamics during natural urination (Figure supplement 11A and Methods, lines 470–474, 652-655). Dual-projecting neurons exhibited robust activation throughout the entire voiding phase that was tightly correlated with intravesical pressure rise and EUS bursting (Figure supplements 11A–11H). However, technical limits of current retrograde tools preclude selective isolation of single-projecting (SPN-only or DGC-only) subsets for independent fiber-photometry recordings and injection restricted to one target unavoidably labels both single- and dual-projecting cells. We now state this technical limitation explicitly (Discussion, lines 426-430).

Accordingly, in the revised Discussion (lines 389-406), we integrate fiber-photometry Ca^2+^ signals with single-unit data from opto-tagged recordings to propose several testable, non-mutually-exclusive models for how dual- and single-projecting PMC^ESR1+^ neurons are engaged during natural urination: “Based on population dynamics obtained by fiber photometry (Figures 1D-1H, Figure supplements 1A-1F, and Figure supplements 11A-11H) and single-neuron firing properties recorded via optrode (Figures 1A-1C), we propose several mechanistic models for the engagement of dual- and single-projecting PMC^ESR1+^ neurons during natural micturition. One possibility is that all three populations (dual-projecting, SPN-projecting and DGC-projecting neurons) are co-activated, with the dual-projecting subset acting as a “bridging amplifier” that sustains rising bladder pressure while coordinating EUS relaxation. Alternatively, SPN-projecting neurons may be recruited first to initiate bladder contraction, followed by DGC-projecting neurons that evoke EUS bursting and facilitate urine entry into the urethra; once flow begins, the urethro-detrusor facilitative reflex could recruit dual-projecting neurons to further enhance voiding efficiency. In addition, contextual or state-dependent urination—such as scent-marking behavior characterized by multiple voiding events with smaller volumes than reflexive urination—may predominantly rely on sequential and cooperative activation of single-projecting neurons. Other recruitment sequences remain conceivable. Future studies combining diverse urination-related behavioral paradigms with simultaneous recordings from projection-specifically labeled PMC neurons will be required to validate and refine these models.”

**Reviewer #3 (Public review):**

Summary:

The paper by Li et al explored the role of Estrogen receptor 1 (Esr1) expressing neurons in the pontine micturition center (PMC), a brainstem region also known as Barrington's nucleus (Hou et al 2016, Keller et al 2018). First, the author conducted bulk Ca2+ imaging/unit recording from PMCESR1 to investigate the correlations of PMCESR1 neural activity to voiding behavior in conscious mice and bladder pressure/external urethral muscle activity in urethane anesthetized mice. Next, the authors conducted optogenetics inactivation/activation of PMCESR1 to confirm the contribution to the voiding behavior also conducted peripheral nerve transection together with optogenetics activation to confirm the independent control of bladder pressure and urethral sphincter muscle.

We sincerely thank you for providing a thoughtful summary and insightful comments on our study.

Weaknesses:

(1) The study demonstrates that pelvic nerve transection reduces urinary volume triggered by PMC ESR1+ cell photoactivation in freely moving mice. Could the role of pudendal nerve transection also be examined in awake mice to provide a more comprehensive understanding of neural involvement?

Thank you for this valuable suggestion. We conducted an additional experiment to determine the contribution of the pudendal nerve to PMC^ESR1+^ neuron-driven voiding in awake mice. Bilateral pudendal nerve transection (PDNx) reduced the optogenetically evoked urine volume compared with sham-operated controls, yet photoactivation of PMC^ESR1+^ neurons still reliably induced urination after PDNx (new Figure 6). Thus, bilateral integrity of the pudendal nerve is required for efficient PMC^ESR1+^ neuron-driven voiding, most likely by transmitting the signals that entrain rhythmic EUS bursting. These data and experimental details have been incorporated into Figure 6, Results (lines 272–276), and Methods (lines 542–545).

(2) While the paper primarily focuses on PMCESR1+ cells in bladder-sphincter coordination, the analysis of PMCESR1+-DGC/SPN neural circuits - given their distinct anatomical projections in the sacral spinal cord - feels underexplored. How do these circuits influence bladder and sphincter function when activated or inhibited? Also, do you have any tracing data to confirm whether bladder-sphincter innervation comes from distinct spinal nuclei?

Thank you for this critical comment. To determine how PMC^ESR1+^ neurons that target distinct sacral nuclei influence bladder–sphincter coordination, we first focused on the dual-projecting subset in a new experiment (Figures supplement 11 and Methods, lines 470–477, 652-655, 669-673). Dual retrograde virus injections into SPN and DGC selectively labelled PMC dual-projecting neurons, a subset of which are ESR1+. Fiber-photometry recordings showed that these cells were active during bladder contraction and sphincter relaxation (Figure supplements 11E-11H), whereas optogenetic activation reliably initiated urination: bladder pressure rose immediately and was followed by rhythmic EUS bursting (Figure supplements 11I-11N and 12B; Results, lines 309-313, 332-335). Thus, the dual-projecting sub-population is sufficient to coordinate bladder contraction with sphincter relaxation. Current retrograde tools do not allow selective isolation of single-projecting (SPN-only or DGC-only) subsets; injecting only one target unavoidably labels both single- and dual-projecting cells. Consequently, we cannot yet compare the functional impact of pure SPN-only versus DGC-only PMC populations. This limitation is now stated explicitly in the revised Discussion (lines 426–430).

In our 2025 paper (Yan et al., Commun Biol, 2025, PMID: 40259086), we used PRV-based retrograde tracing to show that SPN and DGC constitute two separate spinal nuclei controlling the bladder and the EUS, respectively. Classic studies have reached the same conclusion (Yao et al., Nat Neurosci, 2018, PMID: 30361547; Karnup & De Groat, IBRO Reports, 2020, PMID: 32775758; Karnup, Auton Neurosci, 2021, PMID: 34391124). These citations and a concise summary have been added to the Results (lines 289–294).

(3) Although the paper successfully identifies the physiological role of PMCESR1+ cells in bladder-sphincter coordination, the study falls short in examining the electrophysiological properties of PMC ESR1+-DGC/SPN cells. A deeper investigation here would strengthen the findings.

Thank you for this thoughtful suggestion. While a detailed electrophysiological characterization of PMC^ESR1+-DGC/SPN^ neurons would provide complementary information, the primary goal of the present study was to define the in vivo functional dynamics and behavioral role of these neurons during natural urination. As you suggested, further electrophysiological analysis of PMC^ESR1+-DGC/SPN^ neurons will be an important direction for our future work.

(4) The parameters for photoactivation (blue light pulses delivered at 25 Hz for 15 ms, every 30 s) and photoinhibition (pulses at 50 Hz for 20 ms) vary. What drove the selection of these specific parameters? Moreover, for photoactivation experiments, the change in pressure (ΔP = P5 sec - P0 sec) is calculated differently from photoinhibition (Δpressure = Ppeak - Pmin). Can you clarify the reasoning behind these differing approaches?

Thank you for this opportunity to clarify our experimental design. The photoactivation protocol (25 Hz, 15 ms pulses) was chosen because PMC^ESR1+^ neurons faithfully follow this frequency without depolarisation block and it reliably triggers voiding (Keller et al., Nat Neurosci, 2018, PMID:30104734). For photoinhibition we originally stated “50 Hz, 20 ms pulses”, but this was an error. Consistent with the same study (Keller et al., Nat Neurosci, 2018, PMID:30104734), we used continuous light (constant illumination) to maintain sustained suppression. The Methods section has been corrected (lines 659-661, 690-691).

The ΔP formula was tailored to the temporal profile of each manipulation. For activation, ΔP (P_5 sec_ - P_0 sec_) captures the rapid pressure rise after light onset; the same window was used in (Hou et al., Cell. 2016, PMID: 27662084). For inhibition, because saline infusion produces rhythmic reflex voiding, we delivered light at the onset of EUS bursting (i.e. when pressure was already at ~peak). Inhibition abruptly stops the bladder contraction, so the bladder cannot return to its pre-void baseline. The Δpressure (P_peak_ – P_min_) was therefore used to quantify the extent to which the ongoing pressure wave was aborted by photoinhibition. P_min_ is the lowest value reached before the next infusion-driven upswing, making the metric insensitive to the slow baseline drift produced by continuous infusion. These clarifications have been added to the Methods (Methods, lines 676-677, 679-680, 692-693).

(5) The discussion could further emphasize how PMCESR1+ cells coordinate bladder contraction and sphincter relaxation to control urination, highlighting their central role in the initiation and suspension of this process.

Thank you for this valuable comment. We have revised the Discussion to emphasize that PMC^ESR1+^ neurons coordinate urination by sequentially driving bladder contraction followed by sphincter relaxation through their dual projections to the SPN and DGC. We also emphasized that this coordination is essential for the initiation and effective execution of voiding (Discussion, lines 369-388). In addition, in the revised Discussion (Discussion, lines 389-406), we integrate fiber-photometry Ca^2+^ signals with single-unit data from opto-tagged recordings to propose several testable, non-mutually-exclusive models for how PMC^ESR1+^ cells are engaged during natural urination.

(6) In Figure 8, The authors analyze the temporal sequence of bladder pressure and EUS bursting during natural voiding and PMC activation-induced voiding. It would be acceptable to consider the existence of a lower spinal reflex circuit, however, the interpretation of the data contains speculation. Bladder pressure measurement is hard to say reflecting efferent pelvic nerve activity in real time. (As a biological system, bladder contraction is mediated by smooth muscle, and does not reflect real-time efferent pelvic nerve activity. As an experimental set-up, bladder pressure measurement has some delays to reflect bladder pressure because of tubing, but EUS bursting has no delay.) Especially for the inactivation experiment, these factors would contribute to the interpretation of data. This reviewer recommends a rewrite of the section considering these limitations. Most of the section is suitable for the results.

We agree with the reviewer that bladder pressure, mediated by smooth muscle contraction, provides an indirect measure of efferent pelvic nerve activity and is subject to both physiological and experimental delays. Regarding potential delay from the tubing system, pressure propagates in fluid at approximately 1000 m/s (Kela & Pekka, Proceedings of World Academy of Science Engineering & Technology, 2009, DOI: 10.5281/zenodo.1080526). Given that the total tubing length in our setup is 0.5-1 meter, this gives an estimated transmission delay of only 0.5-1 ms. However, this delay is negligible compared with the observed time difference (~700 ms) between the cessation of EUS bursting and the termination of bladder contraction. Theoretically, pressure transmission is not expected to introduce a temporal delay. However, we cannot exclude the possibility that the pressure measurement itself may impose such a delay, because bladder pressure does not necessarily reflect efferent pelvic nerve activity in real time. Future studies using simultaneous recordings of bladder pressure and pelvic nerve discharges will help clarify whether a true temporal delay exists. Nevertheless, we agree that additional physiological or peripheral factors may also contribute to this difference in timing. As suggested by the reviewer, we have revised the discussion to consider the potential influence of other factors, such as urethra-detrusor facilitative reflex (Results, lines 343-349).

**Reviewer #3 (Recommendations for the authors):**

(1) In opto-tag experiments, a comparison of average AP waveform during behavior and during light stimulation should be included as criteria. It should be mostly the same waveform.

Thank you for bringing this to our attention. We have now added this comparison as an inclusion criterion in the revised manuscript. Figure supplement 3B shows representative examples of the average waveforms, and Figure supplement 3C displays the distribution of correlation coefficients between spontaneous and light-evoked spikes for all recorded PMC^ESR1+^ units, all of which exhibited r > 0.8.

(2) Optical fiber implantation seems to be done in two different methods. In Figure 1 and Figure 2, the fiber tip is positioned just above PMC but in Figure 3 it seems to be angled. The information should be included in the Methods section.

Thank you for this important comment. We have now clarified in the Methods that for Figures 1 and 2, the optical fibers were implanted vertically above the PMC, whereas for Figure 3, the left optical fiber was implanted at a 33° lateral angle targeting the PMC (Methods, lines 499-503).

(3) In the closed-loop inhibition experiments of Figure 2, the parameters to start closed-loop photo-inactivation were not described in the method. If it is a manual closed loop, it should be described clearly.

Thank you for raising this important point. We apologize for omitting these details in the original Methods. We have now added a complete description of the manual closed-loop photo-inhibition protocol, including the triggering criteria and operator-controlled timing, in the revised Methods section (lines 602–605).

(4) In Figure 7A/E the authors provide a spinal cord image to show the injection site, but the image is misleading. The figure only shows AAV-infected CRH/ESR1 neurons in the spinal cord section. It does not indicate the AAV injection site or the terminal distribution.

Thank you for your important comment. We apologize for providing a spinal cord image that did not accurately depict the injection site. To rigorously verify that our spinal injections were confined to SPN or DGC, we performed new retrograde-tracing experiments in ESR1-Cre and CRH-Cre mice. A mixture of AAV-Retro-DIO-mCherry or AAV-Retro-DIO-EGFP with the retrograde tracer CTB-647 was injected specifically into SPN or DGC. Only animals in which CTB-647 fluorescence was strictly limited to the target nucleus, without spread to the adjacent region, were included (new Figures 7A and 7E). These data confirmed our original observations and have been pooled in Figure 7. The manuscript and figure have been updated accordingly (Results, lines 297-301, 304-306; Methods, lines 465–466).
